# Neuroprotective effects of rTMS in chronic insomnia: is glymphatic system modulation the key player?

**DOI:** 10.1093/sleep/zsaf084

**Published:** 2025-03-22

**Authors:** Giuseppe Lanza, Maria P Mogavero, Raffaele Ferri

**Affiliations:** Clinical Neurophysiology Research Unit and Sleep Research Centre, Oasi Research Institute-IRCCS, Troina, Italy; Department of Surgery and Medical-Surgical Specialties, University of Catania, Catania, Italy; Vita-Salute San Raffaele University, Milan, Italy; Sleep Disorders Center, Division of Neuroscience, San Raffaele Scientific Institute, Milan, Italy; Clinical Neurophysiology Research Unit and Sleep Research Centre, Oasi Research Institute-IRCCS, Troina, Italy

The glymphatic system, first described by the group led by Maiken Nedergaard [1], is a network responsible for clearing metabolic waste from the brain by facilitating cerebrospinal fluid (CSF) flow through brain tissue. This process is widely believed to be most active during sleep, particularly during non-rapid eye movement stage 3, when slow-wave activity enhances the convective exchange between CSF and interstitial fluid [2, 3]. In this context, the importance of sleep’s homeostatic function and its role in neuroinflammation and maladaptive plasticity in various neurological and neuropsychiatric conditions, particularly neurodegenerative disorders, has recently been highlighted [4, 5]. Moreover, sleep disturbances in cognitive and depressive disorders are no longer viewed merely as a consequence but may also play a pathogenic or contributing role, including at the level of transcranial magnetic stimulation (TMS), as recently reviewed [6]. However, the precise role of the glymphatic system during sleep remains a subject of debate, as recent research has presented conflicting findings regarding its activation [7, 8]. These inconsistencies highlight the need for further research into the conditions that optimize glymphatic function.

The manuscript titled “Enhancement of Glymphatic Function and Cognition in Chronic Insomnia Using Low-Frequency rTMS” [9] contributes to this ongoing discussion by exploring the relationship between chronic insomnia, glymphatic clearance dysfunction, and cognitive function. The study investigates whether low-frequency repetitive transcranial magnetic stimulation (LF-rTMS) can enhance glymphatic clearance and cognitive function in individuals with chronic insomnia. Repetitive TMS is a widely used, safe, and noninvasive neuromodulatory technique [10, 11]. When applied in sleep medicine, the main hypothesis explaining its effects concerns the modulation of synaptic plasticity and the strengthening of connections between brain areas involved in sleep disorders, as typically seen in patients with chronic insomnia, restless legs syndrome, or rapid eye movement (REM) sleep behavior disorder (RBD) [12–14]. Recently, there has been a significant increase in published rTMS studies on primary sleep disorders. A multi-database search supports the evidence that rTMS is safe and effective for chronic insomnia, particularly through bilateral stimulation of the dorsolateral prefrontal cortex, the right parietal cortex, and the dominant primary motor cortex, leading to reduced subjective symptoms and severity scale scores, with effects lasting for weeks [15].

Briefly, findings from the study here commented indicate that patients with chronic insomnia exhibit significantly lower diffusion tensor imaging along the perivascular space (DTI-ALPS) index values compared to healthy controls, suggesting reduced glymphatic clearance efficiency. Furthermore, a negative correlation between insomnia severity and glymphatic function was observed, reinforcing the idea that persistent sleep disturbances may impair this critical waste-clearing mechanism. Additionally, cognitive impairments related to attention, working memory, and inhibitory control were found to be associated with reduced glymphatic clearance efficiency, suggesting a possible link between glymphatic dysfunction and the cognitive decline seen in chronic insomnia [16]. Following ten sessions of LF-rTMS, participants experienced notable improvements in sleep quality and cognitive performance. Importantly, the DTI-ALPS index, a marker of glymphatic function, showed a significant increase three months after treatment, suggesting that neuromodulation can enhance glymphatic clearance. The correlation between improved glymphatic function and better sleep and cognitive performance underscores the potential therapeutic benefits of LF-rTMS. These findings raise intriguing possibilities regarding neuromodulation’s role in mitigating neuroinflammatory and neurodegenerative processes associated with poor sleep. By enhancing glymphatic clearance, LF-rTMS may serve as a novel intervention for conditions where impaired waste clearance contributes to disease progression, such as Alzheimer’s disease [3, 17].

This study provides clinical evidence that chronic insomnia may impair glymphatic clearance, aligning with prior animal model research that suggests glymphatic efficiency is highest during deep sleep [18]. While the reduction in glymphatic function observed in chronic insomnia patients [19] supports the theory that sleep is critical for waste clearance, the fact that LF-rTMS improved glymphatic function after treatment rather than simply during sleep itself suggests a more dynamic and modifiable system than previously assumed. This finding contributes to the ongoing debate on whether glymphatic system activity is strictly tied to sleep or if other factors, such as neural excitability and cerebrovascular function, play an equally significant role in its regulation [8]. In this context, it is known that cortical and/or subcortical chronic cerebrovascular lesions may affect the response rate to rTMS (potentially leading to partial or total resistance to rTMS) and, consequently, impact glymphatic function, as recently reported in patients with “vascular depression” [15]. However, in their study [9] the authors correctly recruited participants without any brain lesions in order to minimize the potential effects of vascular burden on clinical outcomes and glymphatic clearance function.

The study also opens avenues for further research. Understanding the precise mechanisms by which LF-rTMS influences glymphatic function could help refine neuromodulation strategies for neurological disorders linked to impaired clearance pathways. Longitudinal clinical and neurophysiological studies exploring whether LF-rTMS provides lasting glymphatic and cognitive benefits, particularly in populations at risk for neurodegenerative diseases (e.g. individuals with RBD at risk for Parkinsonian syndromes) [20], are necessary. Additionally, alternative interventions such as pharmacological agents or lifestyle modifications, in conjunction with LF-rTMS, may provide an even more effective means of enhancing glymphatic clearance and cognitive resilience. Lastly, a translational avenue of clinical and research interest may be the impact of new orexin receptor antagonists for treating insomnia on TMS-induced therapy responses [21]. In short, the significance of this research [9] may extend beyond sleep medicine, representing a substantial step toward the further integration of individually tailored neuromodulatory techniques in neurology and medicine.

In conclusion, the findings of this study provide valuable insights into the complex relationship between sleep, the glymphatic system, and cognitive function. While the glymphatic system’s activity during sleep remains a contentious topic, evidence suggests that interventions beyond sleep itself, such as LF-rTMS, can significantly impact glymphatic clearance. This underscores the importance of further research into the modulation of brain clearance mechanisms and their potential role in preventing neurodegenerative diseases. The study not only reinforces the link between chronic insomnia and glymphatic dysfunction but also highlights the therapeutic potential of neuromodulation in restoring cognitive function and promoting brain health.

## Data Availability

None declared.

## References

[1] Iliff JJ , Wang M, Liao Y, et al A paravascular pathway facilitates CSF flow through the brain parenchyma and the clearance of interstitial solutes, including amyloid beta. Sci Transl Med. 2012;4(147):147ra111. doi: https://doi.org/10.1126/scitranslmed.3003748 PMC3551275 22896675

[2] Reddy OC , van der Werf YD. The sleeping brain: harnessing the power of the glymphatic system through lifestyle choices. Brain Sci. 2020;10(11):868. doi: https://doi.org/10.3390/brainsci10110868 33212927 PMC7698404

[3] Wafford KA. Aberrant waste disposal in neurodegeneration: why improved sleep could be the solution. Cereb Circ Cogn Behav. 2021;2(100025):100025. doi: https://doi.org/10.1016/j.cccb.2021.100025 36324713 PMC9616228

[4] Papa M , De Luca C, Petta F, Alberghina L, Cirillo G. Astrocyte-neuron interplay in maladaptive plasticity. Neurosci Biobehav Rev. 2014;42(35):35–54. doi: https://doi.org/10.1016/j.neubiorev.2014.01.010 24509064

[5] Morara S , Colangelo AM, Provini L. Microglia-induced maladaptive plasticity can be modulated by neuropeptides in vivo. Neural Plast. 2015;2015(135342):135342. doi: https://doi.org/10.1155/2015/135342 26273481 PMC4529944

[6] Lanza G , Fisicaro F, Dubbioso R, et al A comprehensive review of transcranial magnetic stimulation in secondary dementia. Front Aging Neurosci. 2022;14(995000):995000. doi: https://doi.org/10.3389/fnagi.2022.995000 36225892 PMC9549917

[7] Miao A , Luo T, Hsieh B, et al Brain clearance is reduced during sleep and anesthesia. Nat Neurosci. 2024;27(6):1046–1050. doi: https://doi.org/10.1038/s41593-024-01638-y 38741022 PMC11156584

[8] Christensen J , Yamakawa GR, Shultz SR, Mychasiuk R. Is the glymphatic system the missing link between sleep impairments and neurological disorders? Examining the implications and uncertainties. Prog Neurobiol. 2021;198(101917):101917. doi: https://doi.org/10.1016/j.pneurobio.2020.101917 32991958

[9] Zhang C , Zheng Y, Jiang G, et al Enhancement of glymphatic function and cognition in chronic insomnia using low-frequency rTMS. Sleep. 2025;48(6):1–15. doi: https://doi.org/10.1093/sleep/zsaf083. 40121525

[10] Burke MJ , Fried PJ, Pascual-Leone A. Transcranial magnetic stimulation: neurophysiological and clinical applications. Handb Clin Neurol. 2019;163(73):73–92. doi: https://doi.org/10.1016/B978-0-12-804281-6.00005-7 31590749

[11] Kan RLD , Padberg F, Giron CG, et al Effects of repetitive transcranial magnetic stimulation of the left dorsolateral prefrontal cortex on symptom domains in neuropsychiatric disorders: a systematic review and cross-diagnostic meta-analysis. Lancet Psychiatry. 2023;10(4):252–259. doi: https://doi.org/10.1016/S2215-0366(23)00026-3 36898403

[12] Ma H , Lin J, He J, Lo DHT, Tsang HWH. Effectiveness of TES and rTMS for the treatment of insomnia: meta-analysis and meta-regression of randomized sham-controlled trials. Front Psychiatry. 2021;12:744475. 10.3389/fpsyt.2021.744475 PMC8569107 34744835

[13] Mogavero MP , Mezzapesa DM, Savarese M, DelRosso LM, Lanza G, Ferri R. Morphological analysis of the brain subcortical gray structures in restless legs syndrome. Sleep Med. 2021;88(74):74–80. doi: https://doi.org/10.1016/j.sleep.2021.10.025 34740168

[14] Figorilli M , Lanza G, Congiu P, et al Neurophysiological aspects of REM sleep behavior disorder (RBD): a narrative review. Brain Sci. 2021;11(12):1588. doi: https://doi.org/10.3390/brainsci11121588 34942893 PMC8699681

[15] Lanza G , Fisicaro F, Cantone M, et al Repetitive transcranial magnetic stimulation in primary sleep disorders. Sleep Med Rev. 2023;67(101735):101735. doi: https://doi.org/10.1016/j.smrv.2022.101735 36563570

[16] Zhang X , Yin J, Sun X, Qu Z, Zhang J, Zhang H. The association between insomnia and cognitive decline: a scoping review. Sleep Med. 2024;124(540):540–550. doi: https://doi.org/10.1016/j.sleep.2024.10.021 39447528

[17] Voumvourakis KI , Sideri E, Papadimitropoulos GN, et al The dynamic relationship between the glymphatic system, aging, memory, and sleep. Biomedicines. 2023;11(8):2092. doi: https://doi.org/10.3390/biomedicines11082092 37626589 PMC10452251

[18] Xie L , Kang H, Xu Q, et al Sleep drives metabolite clearance from the adult brain. Science. 2013;342(6156):373–377. doi: https://doi.org/10.1126/science.1241224 24136970 PMC3880190

[19] Xiong R , Feng J, Zhu H, et al Evaluation of glymphatic system dysfunction in patients with insomnia via diffusion tensor image analysis along the perivascular space. Quant Imaging Med Surg. 2025;15(2):1114–1124. doi: https://doi.org/10.21037/qims-24-1447 39995712 PMC11847175

[20] Lanza G , Aricò D, Lanuzza B, et al Facilitatory/inhibitory intracortical imbalance in REM sleep behavior disorder: early electrophysiological marker of neurodegeneration? Sleep. 2020;43(3). doi: https://doi.org/10.1093/sleep/zsz242 31599326

[21] Mogavero MP , Silvani A, Lanza G, DelRosso LM, Ferini-Strambi L, Ferri R. Targeting orexin receptors for the treatment of insomnia: from physiological mechanisms to current clinical evidence and recommendations. Nat Sci Sleep. 2023;15:17–38. doi: https://doi.org/10.2147/NSS.S201994 36713640 PMC9879039

